# A Very Rare Cause of Lower Limb Ischemia in Young People: Popliteal Artery Entrapment

**DOI:** 10.2174/1874192401812010018

**Published:** 2018-03-30

**Authors:** Hasan Baki Altinsoy, Ozkan Alatas, Emjed Khalil, Kenan Abdurrahman Kara, Candan Cudi Okten, Omer Faruk Dogan

**Affiliations:** 1 Department of Radiology, Health Sciences University, Elazig Research and Training Hospital, Elazig, Turkey; 2 Department of Cardiovascular Surgery, Health Sciences University, Dr. Cengiz Aslan Research and Training Hospital, Gaziantep, Turkey; 3 Department of Cardiovascular Surgery, Private Camlica Medicana Hospital, Istanbul, Turkey; 4 Department of Cardiovascular Surgery, Health Sciences University, Adana Numune Research and Training Hospital, Adana, Turkey

**Keywords:** Popliteal artery entrapment syndrome, Diagnosis, Surgery, Treatment

## Abstract

**Background:**

Popliteal artery entrapment syndrome (PAES) is a very rare pathology that can cause lower extremity ischemia in healthy young people. Anomalous anatomic relationships between the popliteal artery (PA) and the surrounding musculo-tendinous structures cause PAES. We present 31 patients with PAES in 35 limbs that were treated surgically in our clinic within a 12-year period.

**Patients and Methods:**

From 2001 to 2015, 31 patients (mean age: 32 ± 7.4 years) underwent surgery for PAES. ; 4 patients presented had bilateral PAES. Doppler ultrasonography (US), magnetic resonance angiography (MRA), and conventional angiography were performed as diagnostic procedures. We detected Type I PAES in 4 limbs and Type II PAES in 12 limbs. In the remaining 19 limbs, we diagnosed Type III or Type IV PAES. Simple release of the PA, PA embolectomy and simple release, and the radial artery (RA) patch angioplasty, with or without thromboendarterectomy (TEA), were performed. In 12 limbs, PA continuity was provided by RA interposition.

**Results:**

With the exception of 5 patients, no complications were seen after surgery. Haematoma was detected in 2 patients and local infection in 2 patients. One patient required a revision for recurrent PA thromboembolic event 12 h after surgery. At a median follow- up of 23 months (range: 11-29 months), there were no postoperative complications.

**Conclusions:**

PAES can result in lower limb ischemia due to chronic vascular trauma in young healthy patients. The use of diagnostic tools such as US, a non-invasive method, and MRA are effective diagnostic tools for early diagnosis. With their combined approach, exact and early diagnosis can be achieved. PA release, alone or with arterial bypass using RA, is a viable treatment option when intervention is necessary to prevent limb loss in the early stages of the disease.

## 1 INTRODUCTION

The true incidence of popliteal artery entrapment syndrome (PAES) is unknown. In a review of 20,000 asymptomatic young people and in a study of autopsy specimens, the incidence was reported to range between 0.17 and 3.5%, leading the authors to conclude that only a small proportion of cases are symptomatic [1-3].

PAES is mostly seen in young people with well-developed muscles. Because this pathology is related to the PA and the surrounding tissue of the popliteal musculotendinous structure, its diagnosis can be delayed. Reports of this pathology in the literature are limited to a number of small series [4-7]. The concomitant entrapment of the popliteal vein with the artery has been reported in 7.6% of cases. PAES is a congenital anomaly of muscle or tendon insertion, in relation to the PA, that causes functional occlusion of the artery (7). This entity results from a developmental defect in which the PA passes medial to and beneath the medial head of the gastrocnemius muscle or a slip of that muscle, with consequent compression of the artery. In rare cases, an anomalous fibrous band or the popliteus muscle deep to the medial head of the gastrocnemius is the compressing structure. Radiologic diagnosis and management of patients involved the surgical repair of anomalous anatomical relationship between the PA and the musculotendinous structures responsible for causing functional occlusion of the PA in young people with no risk factors. The aim of surgical management is to release the musculocutaneous structures, and if necessary, to perform a PA embolectomy or bypass procedures to prevent limb loss. The purpose of our review is to report our diagnostic experiences involving 31 patients with PAES, who over a 14-year period were treated surgically at 2 institutions.

## 2 PATIENTS AND METHODS

Over a period of 14 years, from 2001 to 2015, 31 healthy young patients (26 males) underwent surgery for PAES at 2 institutions. PAES was unilateral in 27 patients and bilateral in 4 patients. The ages of the patients ranged from 23 to 49 years, with a mean of 32±7.9 years. The symptoms related to PAES included swelling, pain, paraesthesia, claudication, and rest pain. Preoperative diagnosis of PAES was based on various combinations of radiologic investigations, including duplex ultrasonography (US), magnetic resonance angiography (MRA), and conventional angiography. The demographics and clinical properties of the patients are summarized in (Table **1**).

Patients were examined to determine whether they had any of the following preoperative associated risk factors: smoking history, presence of concomitant disease, hypertension, hyperlipidaemia, diabetes mellitus, chronic renal failure, or ischemic heart disease. Besides a history of smoking, which 22 patients reported to have, none of the patients had a clinical history of these diseases. The main complaints were progressive intermittent claudication and foot coldness during walking or exercise, with symptom duration lasting from 8 months to 2 years.

Various radiologic investigations, including Doppler ultrasonography US with positional stress test, MRA, and traditional angiography were used to diagnose PAES. Doppler ultrasonography US with positional stress test is an important clinical test that involves determining whether or not there are pedal pulses when the patient’s foot is in plantar hyperflexion. Both limbs of all the patients were examined, with the results showing that 25 of the 35 limbs [8] (71.4%) had a positive result. Because the MRA clearly showed the abnormal anatomic relationship between the PA and the head of the musculotendinous anatomy, a repeat MRA was performed to confirm the definitive diagnosis of PAES in all the patients. Angiographic findings in the neutral position consisted of medial deviation of the proximal PA in 18 limbs (51.4%), poststenotic dilatation of the distal PA in 10 limbs (28.5%), and segmental or longitudinal occlusion of the PA in 3 limbs (8.5%). PAES were described by Rich **et al.** [8] Type II, III, and IV in 35 limbs. In our study, Type I was detected in 4 limbs, Type II in 12 limbs, Type III in 13 limbs, and Type IV in 6 limbs. None of the patients were Type V or Type VI. Surgery was recommended to the patients.

## 3 SURGICAL PROCEDURES

All operations were performed under general anesthesia. We preferred a posterior approach to establish clear visualization of the relationships between the artery and musculotendinous structures. After dissection of the neurovascular bundle in the popliteal fossa, we incised through any anomalous insertions or attachments involving the medial head of the gastrocnemius muscle or other bands causing compression of the PA and veins. Musculotendinous section and PA release were performed. In the 4 patients with Type I PAES, simple release of the PA by division of the medial head of gastrocnemius and popliteus muscles and tendons were performed because the vessel remained undamaged. We detected intraluminal PA thrombus in 12 limbs. Therefore, PA embolectomy and simple release of the muscles and tendons was performed.

Because PA was severely fibrotic in the remaining 15 patients who had Type III and Type IV PAES, it was decided that an arterial reconstruction was the best option. We used the radial artery (RA) for arterial reconstruction in all operations. The RA was harvested from the non-dominant hand after applying the Allen Test. A patch angioplasty with thromboendarterectomy was performed in 8 limbs using the RA. The RA interposition was performed in the remaining 7 patients after division of adjacent muscles and tendons because there were irreversible fibrous thickening of the popliteal arterial wall. In patients who were referred to us due to arterial embolic event and for whom an arterial patch plasty or arterial graft interposition was performed, we administered a low molecular weight heparin in the early postoperative period.

The mean size of PA was 3.6 ± 0.9 mm (2.9-5.1 mm) in 12 patients who underwent the RA graft interposition. Because of the RA size was smaller than PA in some patients, we used a mixed vasodilator solution prior to anastomosis. We incubated the RA in vasodilation solution (including a calcium channel inhibitor, papaverine, and nitroglycerine). We previously described the efficasy of this mixed solution [9]. The RAs’ diameter increased significantly after incubation. The mean diameter of the RA was 2.2± 0.4 mm prior to incubation. After incubation, the mean diameter of the RA was measured as 4.06±1.1 mm. Therefore, there was no mismatch between the size of popliteal and the RA.

Postoperatively, antiplatelet and antiaggregant drugs, clopidogrel (daily dose of 75 mg), and acetylsalicylic acid (ASA) (daily dose of 100 mg) were prescribed for each patient after discharge.

## 4 CASE PRESENTATIONS

### 4.1 Case 4

A 23-year-old young woman presented with a 3-year history of experiencing numbness and pain in the right lower leg during exercise. Over the past 2 years, the patient had experienced worsening leg pain when running long distances. The patient had no cardiovascular risk factors. She had been hospitalized in the orthopaedic and physiotherapy clinic due to leg pain prior to admission to our clinic. However, her symptoms continued to persist. Examination of the right leg showed that there was swelling and no pulse below the femoral artery. Pulses were normal in left lower limb. Magnetic resonance imaging (MRI) revealed normal aortic and iliac arteries. Anterior and posterior views of MRA showed an occlusion of the right popliteal and peroneal arteries Figs. (**1** and **2**). In this case, intra-arterial thrombolysis was performed. Control angiography showed that there was a thrombus in the artery and a stricture at the mid-PA, with an irregularity of the wall. At the end of two 2 days, the angiogram demonstrated that the thrombus had largely resolved; however, the stricture of PA was present. MRI scanning was subsequently performed, which showed that the medial head of the gastrocnemius muscle, the PA and vein where it inserted in a more lateral position Fig. (**3**). Further evidence from the MRI revealed that the PA crossed below the popliteal muscle in this patient with Type IV PAES. Contralateral limb MRI was normal. Posterior popliteal approach was preferred. An S-shaped skin incision was performed. The PA crossed under the popliteus muscle and there was a hypertrophic band, which was the cause of occlusive pressure on the PA. The hypertrophic bands and the popliteus muscle were resected. PA was opened and it was partially occluded. The tibioperoneal artery was also occluded. A thromboendarterectomy (TEA) was performed. The artery was repaired using a RA. No rest pain was detected after surgery. US showed that the distal arterial system and the RA were patent. A low molecular weight heparin and acetylsalicylic acid were prescribed in the postoperative period. The patient was discharged home in good clinical condition. We prescribed a daily dose of 100 mg of acetylsalicylic acid. The patient was able to run without any symptoms in the lower limb.

### 4.2 Case 8

A 25-year-old man presented with a 3-year history of pain in the left lower leg during walking. Claudication was the main symptom. The patient had no other risk factors for atherosclerosis or Buerger’s disease. Systemic and cardiac system examinations were normal. All pulses including popliteal and distal arteries were not palpable in the neutral position of left leg. Leg swelling, and ischemic symptoms were detected. There were palpable femoral pulses. Motor and sensory functions of both lower limbs was intact. The patient refused the conventional angiography. Therefore, an MRI scan was performed, which showed the aorta and iliac arteries to be normal. There was an occluded left PA. MRI demonstrated that the PA crossed below the popliteal muscle Fig. (**4**). We suggested an operation due to ischemia of the lower limb, but the patient declined a surgical approach. Therefore medical therapy, which included administration of antiaggregant and anticoagulant drugs, was prescribed. Six months later, this patient presented to our emergency clinic with the complaint of ischemic leg symptoms. Conventional angiography showed that there was a total occlusion of PA because of an acute thrombus. We operated in an emergent situation. The medial head of the gastrocnemius muscle was totally divided, and fibrotic bands were cut. Embolectomy was performed using a Fogarty catheter. PA blood flow was provided using a saphenous vein interposition. Dual antiplatelet treatment, clopidogrel (daily dose of 75 mgr), and acetylsalicylic acid (daily dose of 100 mgr) were prescribed after surgery. The patient was discharged to home in a good clinical condition 5 days after surgery.

### 4.3 Case 11

A healthy 27-year-old male presented to our hospital with claudication in both legs. He was a non-smoker. No paraesthesia, weakness, or other ischemic symptoms were apparent. On physical examination, there was no pulse of the dorsalis pedis. A MRA showed an occluded PA. MRI of the right leg demonstrated that there was severe compression of the PA by an accessory slip of gastrocnemius muscle (Type III PAES) of the right leg Fig. (**5A**). Lateral view of MR imaging of the popliteal region showed the gastrocnemius muscle attached more laterally Fig. (**5B**). Surgery for relief of compressed PA was carried out. A myotomy was performed and fibrotic bands were cut. PA blood flow was provided using a RA interposition. Postoperative angiography showed the complete resolution of the PA compression. The RA graft was patent. The patient was discharged home on postoperative day 6. We prescribed clopidogrel for a year. The patient has no ischemic symptoms.

### 4.4 Case 18

**Fig. (7):**
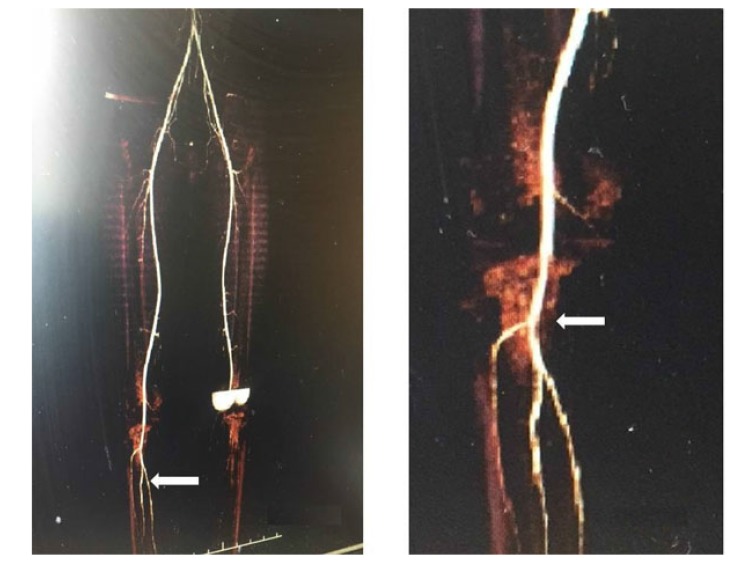
Computed tomography angiography of the abdominal aorta and the distal limb arteries exhibits a patent popliteal, and the anterior tibial, and the peroneal artery 6 months after surgery (white arrowhead).

A 27-year-old professional female athlete was referred to our clinic because of sudden embolic events of the left lower leg. She described severe pain in the left leg during an exertion. The patient was a non-smoker. The bilateral femoral arteries were patent. However, the left PA and the pedal pulse were not palpable. Doppler US showed no colour filling with hyperechoic luminal thrombus. Motor and sensory functions of the legs were normal. A computed tomography (CT) showed an occlusion of the left PA due to intra-arterial thrombus Fig. (**6A**). We performed an intra-arterial thrombolysis using a glycoprotein IIb/IIIa inhibitor (tirofiban) followed by intra-arterial urokinase. Twenty-four hours later, ischaemic symptoms, including pain and pallor, started again on in the right limb. The distal pulses were not palpable. Because of the suspicion of PAES, a MRA was performed. The MRA results showed that total occlusion of the left PA Fig. (**6B**). In this case, the medial head of the gastrocnemius muscle was attached laterally. The PA was crossed under the gastrocnemius muscle insertion which described Type III PAES with the total occlusion of the right PA. Therefore, surgery was recommended. The medial head of the gastrocnemius muscle and tendon were carefully divided, and an embolectomy was performed. The distal pulses were palpable after surgery. The patient had no ischemic symptoms. Clopidogrel was prescribed as an antiaggregant. The patient was discharged from the hospital in good clinical condition. A CT angiography was performed 6 months later demonstrated that the popliteal, anterior tibial, and the peroneal arteries were patent (Fig. **7**).

## 5 DISCUSSION

We presented our diagnostic modalities and treatment strategy for 31 young patients with PAES who were admitted to 2 cardiovascular surgery clinics because of calf pain, cramping during walking, ischemia, and swelling of the affected lower limb. There were 4 female patients (12.9%). For the diagnosis of PAES, we routinely used Doppler US for the first 12 patients with passive plantar flexion and traditional angiography was performed. However, because conventional angiography is not able to show the anatomical relationship between the PA and popliteal venous system (except slight medial deviation of the PA), MRI was the first choice of diagnostic modality after Doppler US. Symptoms were limited to intermittent claudication due to the intermittent compression of the artery during plantar hyper dorsiflexion.

Chronic extrinsic arterial compression leads to vascular endothelial trauma, early arterial wall fibrosis, and subsequent thrombosis. Thrombus formation may cause complete obstruction of the compressed PA, which in turn can lead to leg ischemia due to insufficient collateral circulation of the leg. We therefore performed a thromboembolectomy or TEA and patch plasty in 12 patients. In 15 patients, the RA interposition was performed after TEA. For the first time such a sequence of procedures has been conducted. In the literature, it has been reported that either the great saphenous vein or a 7 or 8 mm polytetrafluoroethylene graft can be used for the construction of the PA.

The the Popliteal Vascular Entrapment Forum in 1998 was an attempt to gain some consensus on the anatomic classification of the different types of PAES. Type I is characterized by an atypical course of the PA, Type II occurs as a result of an abnormal muscular insertion. In Type III, an accessory slip of muscle from gastrocnemius slings around the artery. In patients of Type IV PAES, an artery lies deep in popliteal fossa entrapped by popliteus or fibrous band. In Types V and VI, both PA and vein are entrapped.

Traditional invasive angiography is the standard diagnostic tool for patients with ischemic symptoms. The use of this technique, however, is unable to demonstrate compression of the PA in some patients. Gourgiotis **et al.**, in addition to other authors, have noted that irregularity of the wall of the PA should raise suspicion of PAES [8, 10]. MRI is useful for evaluating aberrant muscular anatomy of the popliteal fossa and also for showing compression and deviation due to peripheral tissue effects on the PA and anomalous insertion of gastrocnemius or popliteal popliteal muscle and tendons [11-15]. Our experiences showed that MRA was useful for detecting the anatomic relationship between the artery and the calf muscles in young males with acute limb ischaemia.

The most common and successful treatment option is to perform a myotomy of popliteal muscle to release the entrapment, an effective treatment for all types of PAES. A resection of the hypertrophic structures should be done. If early diagnosis is not made, fibrosis or total occlusion of the PA could occur. Distal emboli due to poststenotic aneurysm or acute thrombus formation can be seen in patients with PAES [16]. Halici **et al.** reported on 12 patients (9 males, 3 females) with PAES in 2016 [17]. In a study by Meier **et al.**, patients with PAES were successfully treated by intravascular thrombolysis and thromboembolectomy, followed by musculotendinous dissection [18]. The surgical options available to these authors included TEA with patch angioplasty and an autogenous vein graft interposition after segmental arterial resection [17]. Interposition of vein grafting is superior to TEA and vein patching for stenosis, as it has a complication rate of 16.7% compared with 45.5% in the others [18]. In our patients who underwent a RA patch plasty, we did not detect any stenosis during the follow-up. The patency rate of bypass surgery using vein grafts has been reported to be 57-65% over a period of 8-10 years [19, 20]. Levien **et al.** reported a case series of 66 limbs that underwent musculotendinous section and 16 limbs that underwent segmental replacement of the occluded PA with a reversed saphenous vein [21]. The limbs treated using vein grafts demonstrated no graft occlusion. On the other hand, di Marzo **et al.** reported that 15 limbs that underwent revascularization procedures exhibited a patency rate of 65% [22]. Kim **et al.** also reported that bypass surgery was associated with a worse patency rate than interposition procedures [23]. Igari **et al.** suggested that the treatment of PAES with myotomy and selective revascularization achieved good long-term outcomes [24].

Given the seriousness of PAES, particularly as it threatens loss of limbs, clinicians should consider early diagnosis of PAES in young male patients who present with ischemic symptoms. PAES and thromboangiitis obliterans (Buerger’s disease) are both characterized by distal arteriopathy in young males, but the treatment options are different. Patients with Buerger disease are generally heavy smokers. This pathology may sometimes be misdiagnosed as atherosclerosis or Buerger’s disease. Igari **et al.** reported a case with PAES which had been misdiagnosed as Buerger’s disease [25]. There are of course cases of male smokers who present with complaints of bilateral intermittent claudication and are referred from orthopaedic clinics for suspicion of Buerger’s disease. We believe that an MRA and a Doppler US, accompanied by a plantar hyperflexion test, should be done for differential diagnosis and early treatment.

We performed MRA and CT as the main diagnostic procedures for diagnosis of PAES and analysed the relationship between the artery and musculocutaneous tissue in all patients. These modalities can demonstrate the vessel lumen as well as the surrounding anatomy. Traditional angiography was performed in the neutral limbs position, as well as with the foot in either dorsiflexion or plantar flexion to elicit compression and to confirm the diagnosis prior to surgery. Imaging commonly shows a normal arterial lumen when the foot is in the relaxed position, and a narrowing of the arterial lumen during stress manueuvres [26] for differentiation of susceptible arterial thrombosis [27].

PAES is not an indication for angioplasty or stent placement; however, interventional thrombolysis would be appropriate treatment for patients who present with occlusion due to PAES. Thrombolysis of the distal popliteal and interventional thrombolysis would be appropriate therapy for patients who present with acute occlusion of popliteal or distal arterial trees due to PAES [21]. Thrombolysis of the distal popliteal and runoff vessels can be very important prior to surgical correction. In addition, MRI may not provide an adequate information of collateral arterial system of limb arteries. Therefore, to provide collateral circulation system during an angiography, we used conventional angiography to clearly visualise the run off of the limb arteries.

Ankle-brachial index (ABI) has been suggested as a sign of arterial impairment in patients with PAES [28]. In previous publications, the results of post-exercise test with a doppler US/ABI have been reported as a main part of the investigations for PAES prior to treatment [29] [30] [31]. ABI is a simple, noninvasive, effective, and inexpensive method. Collins **et al.**, and Ruppert **et al.** proposed the provocation manoeuvres as a pathway for diagnosis of PAES [29] [30]. In this method, the ABI measurement should be obtained at rest, and just after treadmill using an initial speed of 6 km/h [31]. To produce symptoms, the speed of treadmill may be increased during the exercise. The pressures of exercise compared with the baseline brachial pressures. If there is a reduction in indices, a failure to increase the index with exercise with a normal increase in brachial pressure >5 mm Hg above resting systolic, the examination is accepted as a positive test [30] [31].

PAES is a rare cause of a lower limb -threatening condition in healthy young people. Patients with PAES are reported as small case series in the English literature. As part of this study, we presented our 14-year experience with 31 patients (35 affected limbs). For the first time, we also described the use of the RA for PA reconstruction. If a young patient presents with unexplained lower limb pain experienced during walking, a diagnosis of PAES should be considered. Acute PA embolus can be seen as a first symptom in these patients who have no cardiovascular risk factors. In our opinion, Doppler US, using passive or active dorsiflexion of foot, and MRI should be performed as non-invasive methods for diagnosis of PAES. A posterior approach using an S-shaped incision to establish clear visualization of abnormal insertion of muscles and hypertrophic bands is the best option. If there is arterial fibrosis due to chronic arterial trauma, the fibrotic segment should be resected. The RA may be used as an arterial conduit to provide PA continuity. An antiaggregant such as clopidogrel, and a calcium channel blocker to inhibit RA spasm, can be used for up to 1 year in the post-surgery period.

## Figures and Tables

**Fig. (1):**
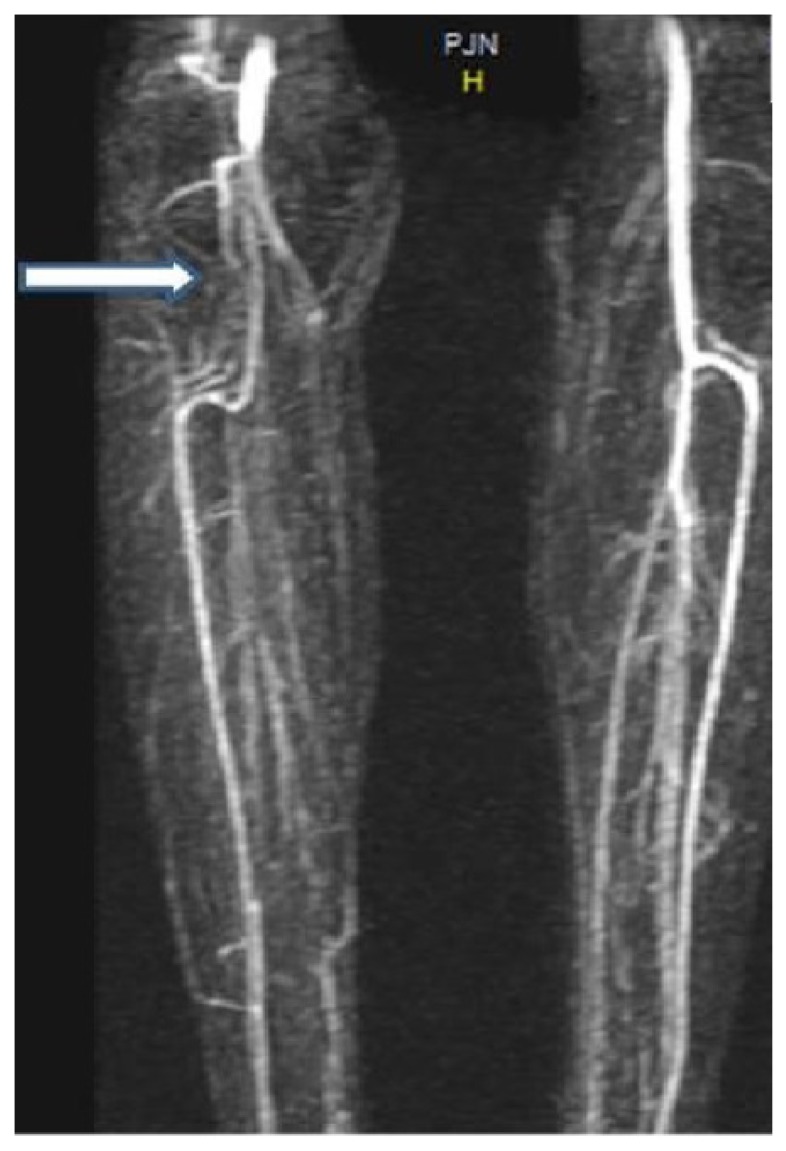
Anterior view of magnetic resonance angiography shows the occlusion of the right popliteal artery due to external compression by the adjacent tissue. There are no signs of atherosclerotic changes in the bilateral lower limb arteries.

**Fig. (2):**
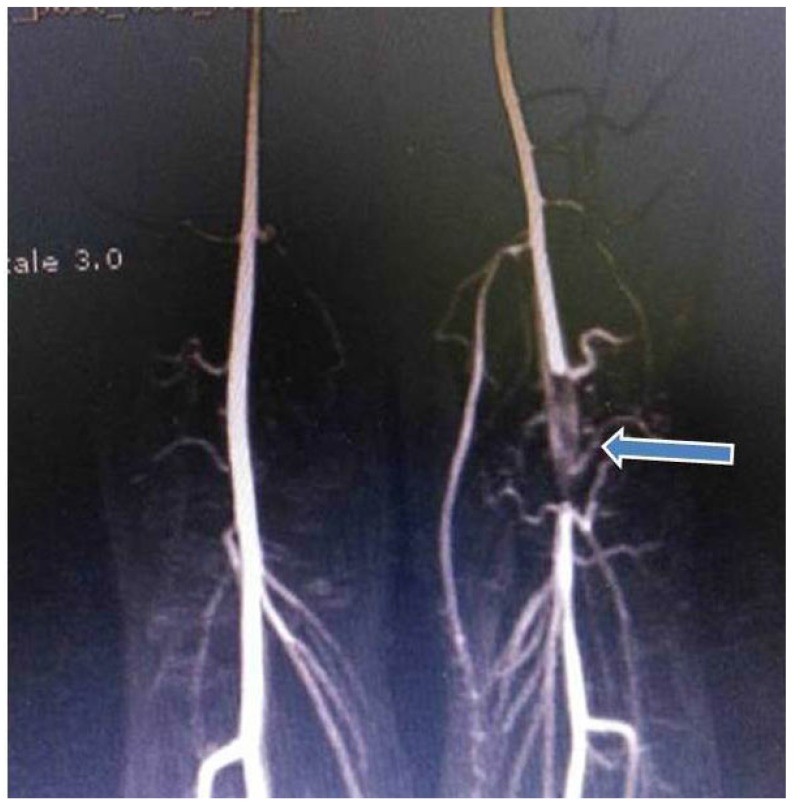
Posterior view of magnetic resonance angiography shows an occluded popliteal artery in the same patient (arrow).

**Fig. (3):**
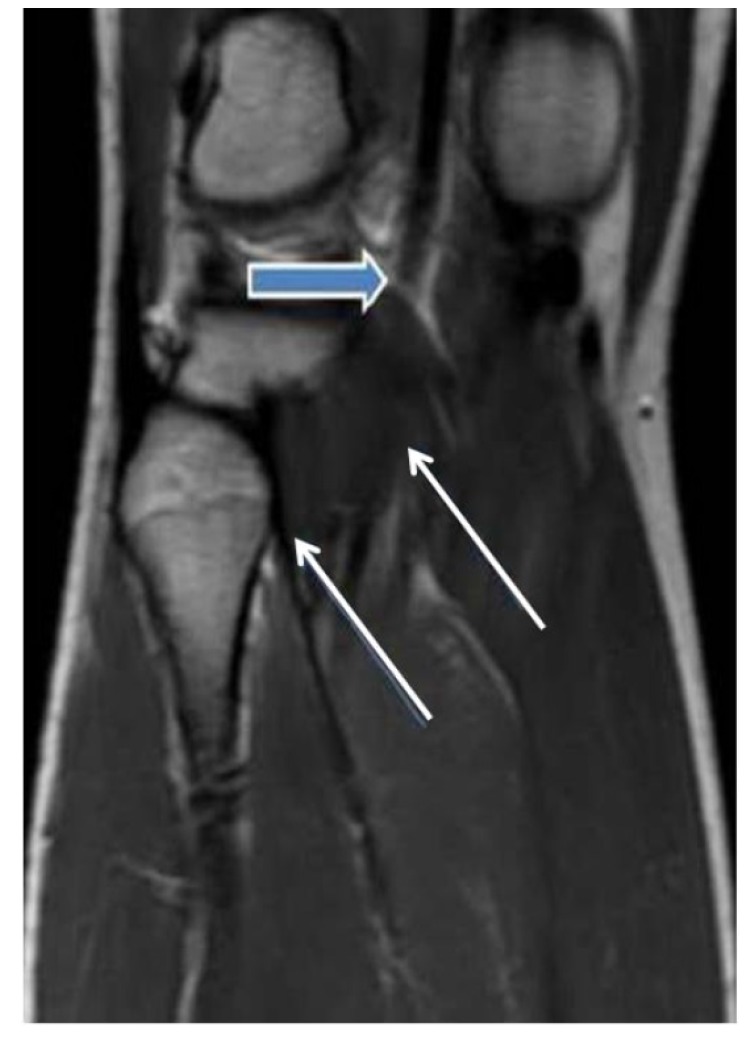
Magnetic resonance imaging shows a popliteal artery entrapment in a 25- year- old patient. T1 weighted axial image demonstrates that the popliteal artery crossed below the popliteal muscle (the white arrows shows the relationship between adjacent tendon and the popliteal artery, while the blue arrow exhibits the relationship between the artery and muscles in the popliteal fossa).

**Fig. (4):**
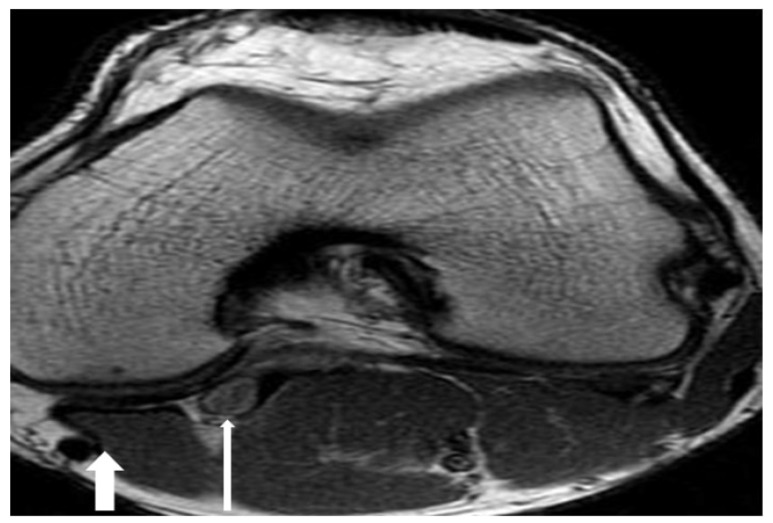
A transverse spin-echo T1-weighted magnetic resonance image shows a compression of popliteal artery by gastrocnemius muscle. The long and the short *white arrows* demonstrates a popliteal artery and an abnormal insertion of muscle, respectively.

**Fig. (5):**
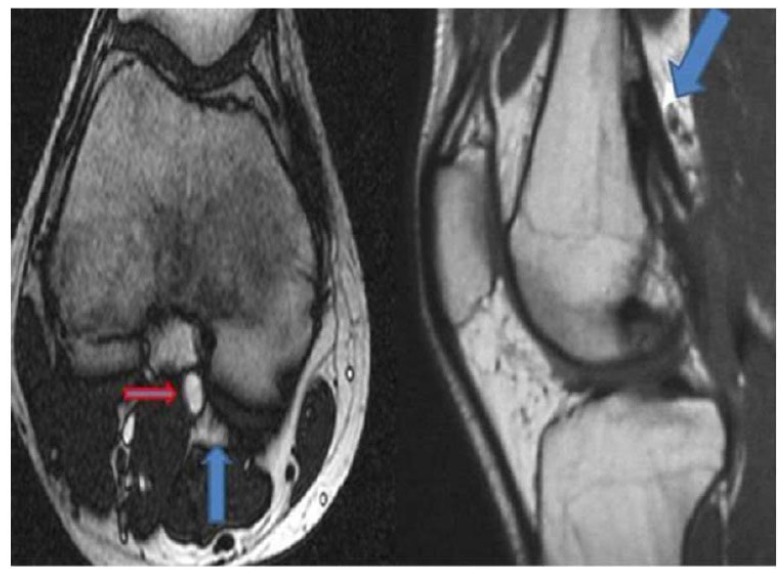
**5A Magnetic resonance imaging** of popliteal region shows a Type III popliteal artery entrapment of the right leg. Compression of popliteal artery by an accessory slip of gastrocnemius muscle is visualized (blue arrow). The red arrow shows the compression of the popliteal artery. **5B** A lateral view of magnetic resonance imaging demonstrates the abnormal insertion of medial head of the gastrocnemius muscle (blue arrow). A slight deviation of the left popliteal artery resulting from the muscle which is attached more laterally. The popliteal artery is crossed under the gastrocnemius muscle insertion (Type III PAES) with total occlusion of the right popliteal artery. The *white arrow* shows a total occlusion of the left popliteal artery.

**Fig. (6):**
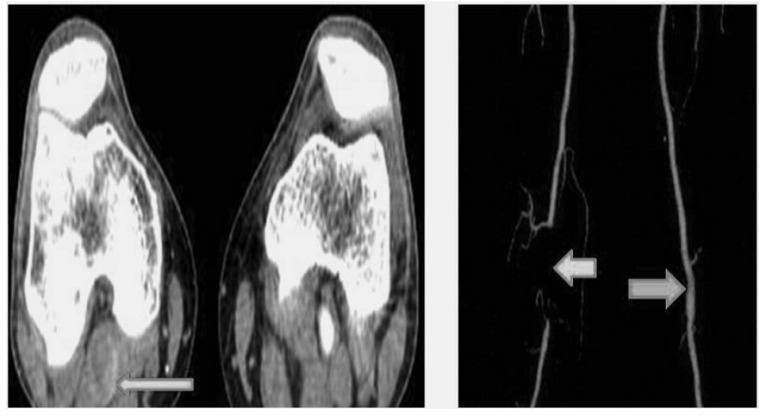
**6A** Bilateral computed tomography of the popliteal region shows a popliteal artery entrapment of the left limb. A total occlusion of the left popliteal artery by an accessory slip of muscle is visualized (white arrow). **6B** A magnetic resonance angiography clearly shows a segmental total occlusion of the right popliteal artery *(white arrow)* resulting from abnormal insertion of medial head of the gastrocnemius muscle.

**Table 1:** Clinical charecteristics of the patients with popliteal artery entrapment syndrome.

Variable	Number
**Sex**	
Male	27
Female	4
**Smoking**	
No	9
Yes	22
**Laterality**	
Right	15
Left	12
Bilateral	4
**Symptoms**	
Intermittent claudication	26
Foot Coldness	11
Calf Pain	9
Swelling	8
